# Tunability of p- and n-channel TiO_x_ thin film transistors

**DOI:** 10.1038/s41598-018-27598-5

**Published:** 2018-06-18

**Authors:** Wu-Chang Peng, Yao-Ching Chen, Ju-Liang He, Sin-Liang Ou, Ray-Hua Horng, Dong-Sing Wuu

**Affiliations:** 1 Department of Materials Science and Engineering, National Chung Hsing University, No. 145, Xingda Road, Taichung, 40227 Taiwan; 2 Department of Materials Science and Engineering, Feng Chia University, No. 100, Wenhwa Road, Taichung, 40724 Taiwan; 3 Department of Materials Science and Engineering, Da-Yeh University, No. 168, University Road, Changhua, 51591 Taiwan; 4 Institute of Electronics, National Chiao Tung University, No. 1001, University Road, Hsinchu, 30010 Taiwan; 5 Innovation and Development Center of Sustainable Agriculture (IDCSA), National Chung Hsing University, No. 145, Xingda Road, Taichung, 40227 Taiwan

## Abstract

To acquire device-quality TiO_x_ films usually needs high-temperature growth or additional post-thermal treatment. However, both processes make it very difficult to form the p-type TiO_x_ even under oxygen-poor growth condition. With the aid of high energy generated by high power impulse magnetron sputtering (HIPIMS), a highly stable p-type TiO_x_ film with good quality can be achieved. In this research, by varying the oxygen flow rate, p-type γ-TiO and n-type TiO_2_ films were both prepared by HIPIMS. Furthermore, p- and n-type thin film transistors employing γ-TiO and TiO_2_ as channel layers possess the field-effect carrier mobilities of 0.2 and 0.7 cm^2^/Vs, while their on/off current ratios are 1.7 × 10^4^ and 2.5 × 10^5^, respectively. The first presented p-type γ-TiO TFT is a major breakthrough for fabricating the TiO_x_-based p-n combinational devices. Additionally, our work also confirms HIPIMS offers the possibility of growing both p- and n-type conductive oxides, significantly expanding the practical usage of this technique.

## Introduction

Great progress in oxide semiconductor-based electronics have been made in this decade^1^. Oxide semiconductors with n-type or p-type characteristics are an interesting class of materials widely studied for optoelectronic applications. Particularly, the all- oxide devices which involves construction of p-n junction from stacking n-type and p-type materials, have also been developed for use in rectifier^2^, near ultraviolet (UV) light-emitting diode (LED)^3^, solar cell^4^, and complementary thin film transistor (TFT)^5^.

In the view of TFT devices, a variety of oxide semiconductors has been demonstrated as suitable materials to fabricate high performance n-channel TFT devices; however, fabrication of a p-channel TFT with equal performance comparable to n-channel TFT is still a crucial challenge^6^. According to the Madelung’s potential theory^7^, the conduction band minimum (CBM) is mainly made of the metal cation and valence band maximum (VBM) of oxygen *2p* orbitals in typical metal oxide materials. The conduction band in n-type oxide semiconductors is mainly derived from large spherically symmetric metal *ns* orbital. The hybridization is hence not limited to the nearest metal cation but even extends to second neighbor metal cation, resulting in very low electron effective mass and maximum electron mobility^6,7^. On the other hand, the carrier conduction path (valence band) in p-type oxide semiconductors is mainly formed from oxygen *p* orbitals. The localized nature of *p*-orbitals leads to large hole effective mass, which severely limits the hole mobility in p-type metal oxides. Such reason results the main difficulty in achieving high performance p-channel oxide semiconductors-based TFTs^6,8^.

Owing to the advantages of low cost, eco-friendliness, and abundance of Ti element, titanium oxide (TiO_x_) materials are extensively explored for potential applications in numerous fields. Basically, TiO_x_ material exists in two stable forms; titanium dioxide (TiO_2_) with oxidation state +4, and titanium monoxide (TiO) with oxidation state +2. TiO_2_ is a well-known n-type semiconductor in un-doped or doped conditions because of the presence of intrinsic defects, i.e., oxygen vacancies^9,10^. Moreover, its unique characteristics including intrinsic defects, wide band gap, and high field-effect carrier mobility, renders TiO_2_ a promising oxide semiconductor as active channel for resistive random access memory^11^ and n-type TFT^12,13^. On the contrary, TiO shows an intrinsic p-type semiconductor in nature^14,15^. As compared with TiO_2_, the dramatic change in the polarity arises from a high amount of structural vacancies up to 10–15 at.% on both the Ti and the O sublattices at a varied stoichiometric composition^14,15^. Therefore, its structural vacancies and stoichiometric ratio play an essential role in the relevant optical, electrical, magnetic and thermal properties^14,15^. With same element oxides possessing both p-type and n-type forms, the polarity change by manipulating the stoichiometric ratio of metal and nonmetal in oxide semiconductors can also be found in tin oxide-based system, i.e., n-type tin dioxide (SnO_2_), and p-type tin monoxide (SnO)^6^.

Though there are reports of n-channel TiO_2_ TFTs^12,13^, very few studies have been reported, if not none, to adopt TiO film as active channel for p-type TFTs. In our previous study^16^, this study demonstrated the feasibility of using high power impulse magnetron sputtering (HIPIMS) to prepare crystalline γ-TiO film. Systematic investigation of the γ-TiO films obtained as a function of substrate bias voltage and post-annealing temperature on microstructural, and optoelectrical properties were carried out. Furthermore, the optimum γ-TiO film showed a p-type characteristic and a high hall hole mobility of 8.2 cm^2^/Vs^16^. Based on the abovementioned results, the aim of this study is to synthesis TiO_x_ film by using HIPIMS technique, and the p-type and n-type for γ-TiO and TiO_2_ films, respectively, were achieved by simply varying oxygen flow rate during the film deposition. Moreover, the performance of both p-type γ-TiO and n-type TiO_2_ TFTs are evaluated and demonstrated. Through the growth of p-type γ-TiO and n-type TiO_2_ by HIPIMS, the applications of TiO/TiO_2_ can become more widely. For example, the fully oxide-based complementary metal–oxide–semiconductors (CMOSs) and the p-n junction optoelectronic devices both fabricated by p-type TiO/n-type TiO_2_ could be achieved by HIPIMS. Most importantly, the tunable conductivity of materials via HIPIMS can be used for other oxides consisting of ZnO, SnO_2_, In_2_O_3_, and so on), expanding the practicality of this growth method.

## Results and Discussion

**Figure 1:**
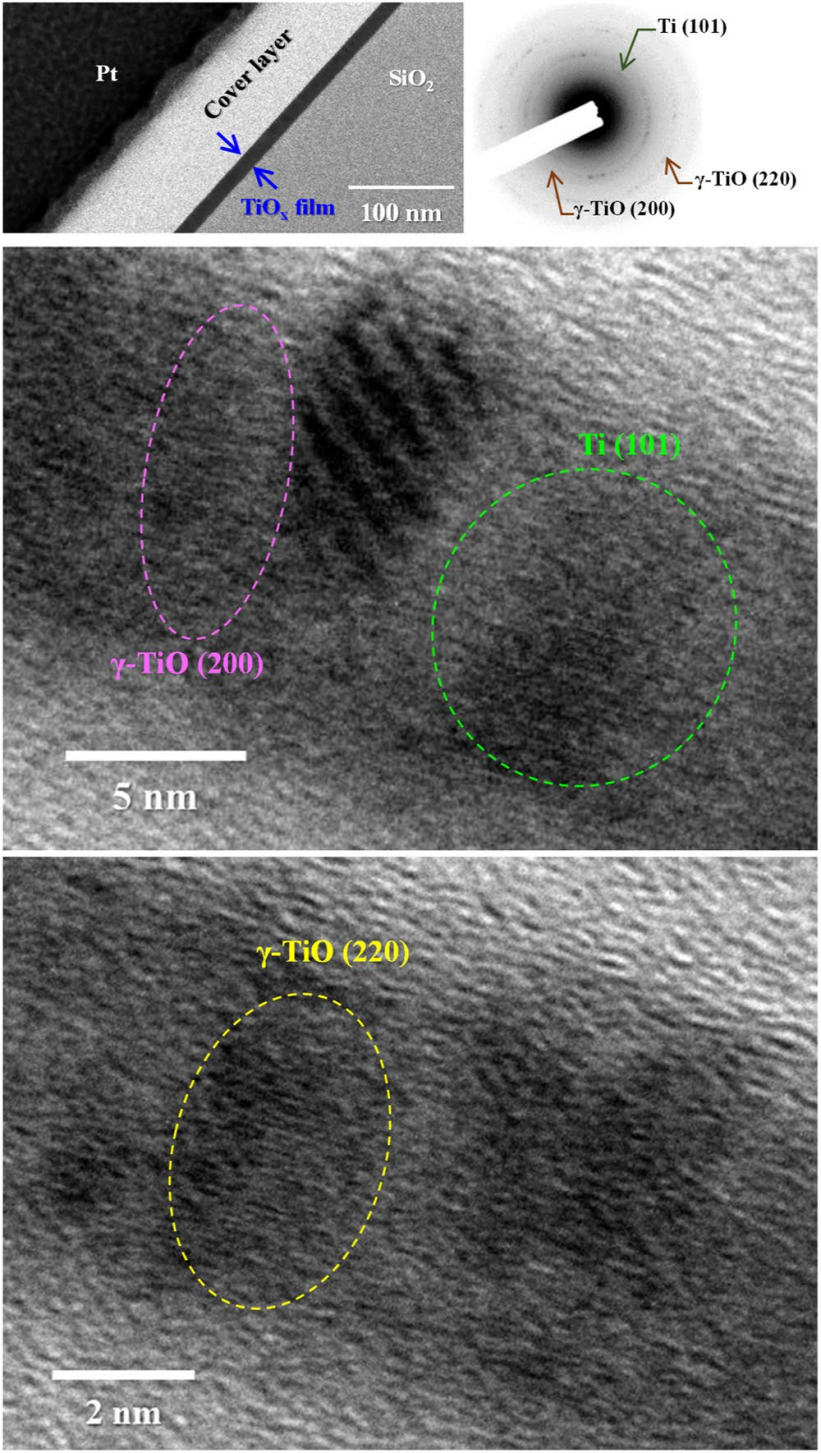
Cross-sectional BF images of the TiO_x_ films deposited by HIPIMS under the oxygen flow rate of 15 sccm. HRTEM images and SAED patterns are also shown in this figure.

**Figure 2:**
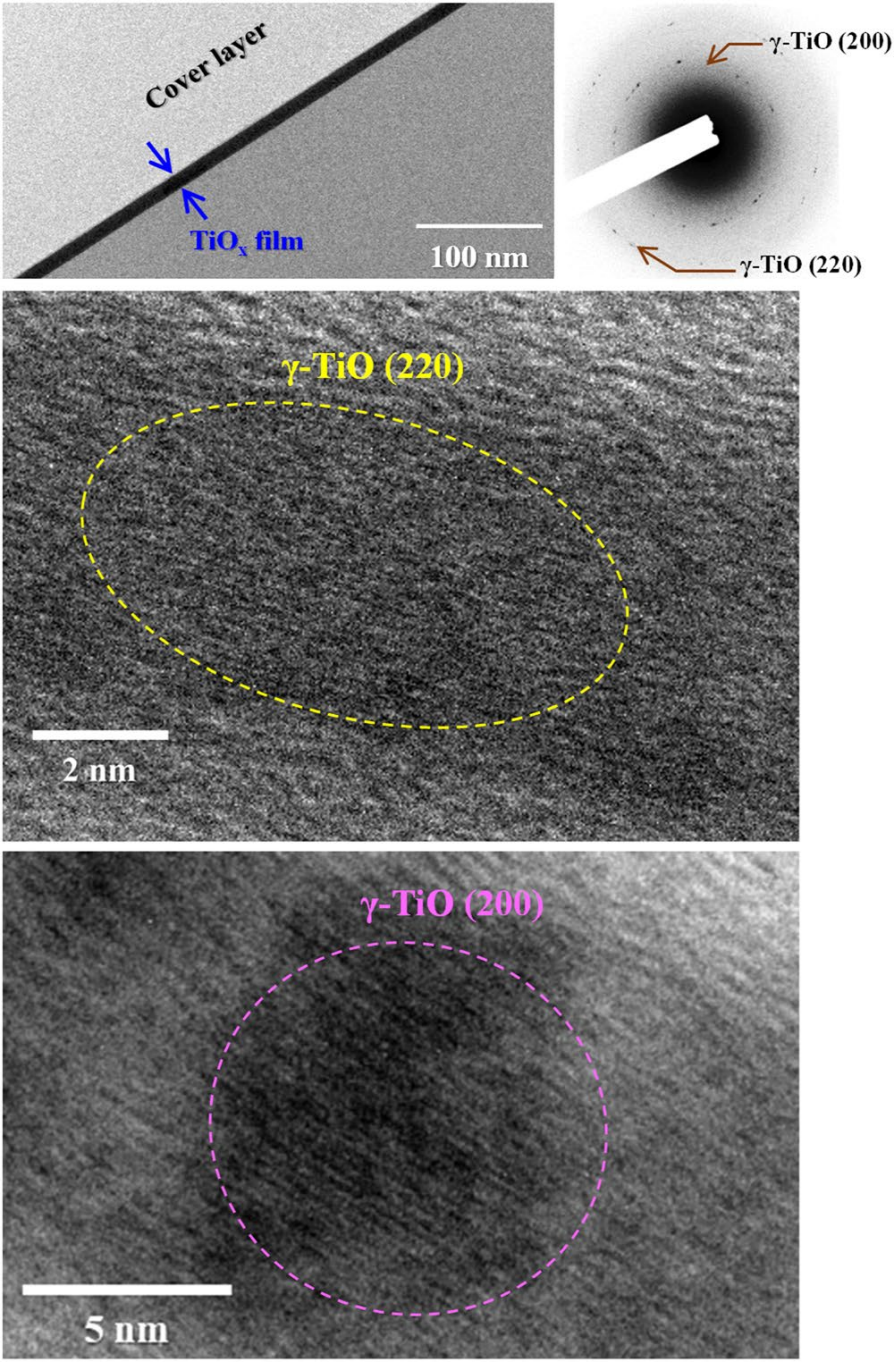
Cross-sectional BF images of the TiO_x_ films deposited by HIPIMS under the oxygen flow rate of 17.5 sccm. HRTEM images and SAED patterns are also shown in this figure.

**Figure 3:**
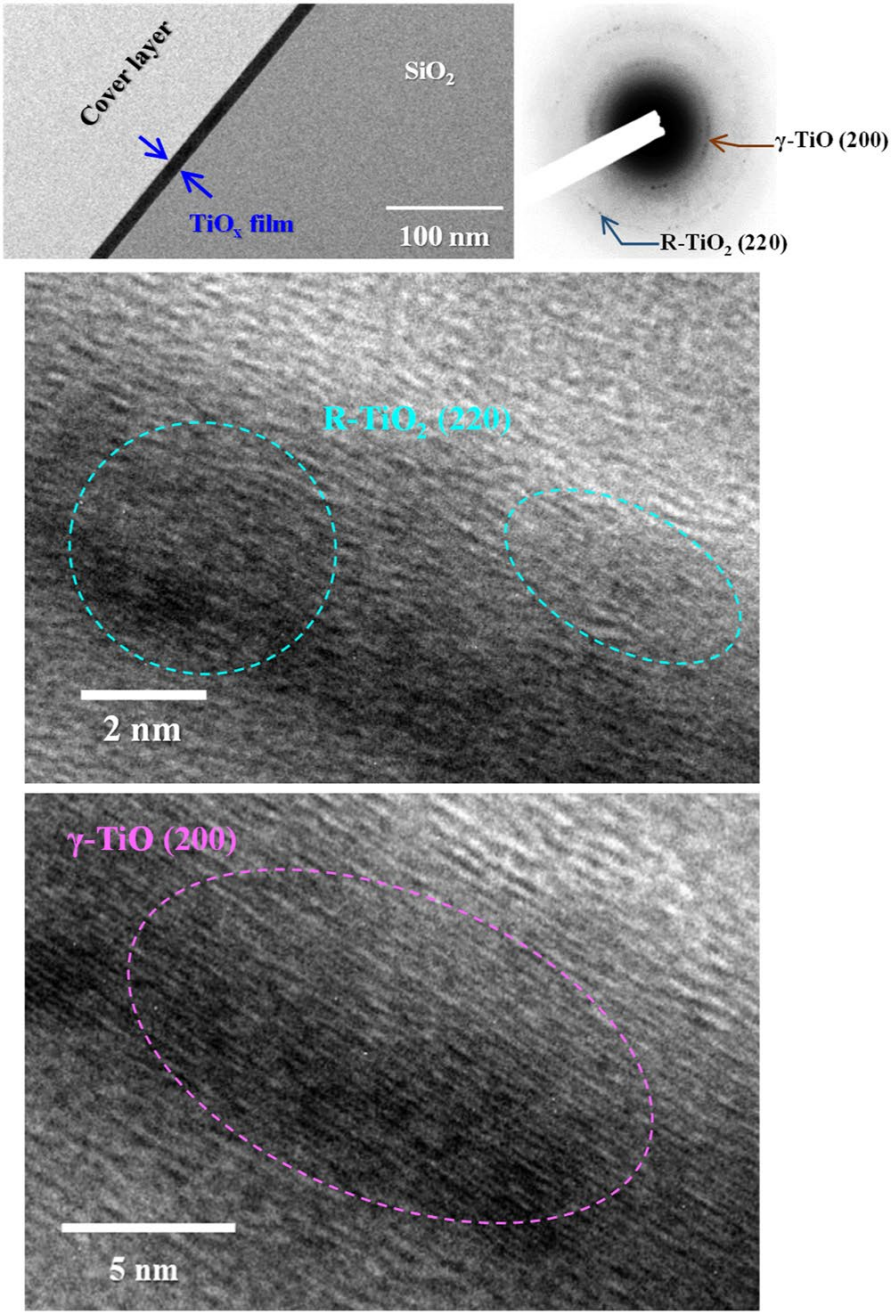
Cross-sectional BF images of the TiO_x_ films deposited by HIPIMS under the oxygen flow rate of 20 sccm. HRTEM images and SAED patterns are also shown in this figure.

To full understand the microstructure of the TiO_x_ films deposited by HIPIMS, TEM study was carried out. Figures 1–3 show the cross-sectional BF images of the TiO_x_ films prepared at different oxygen flow rates, and their high-resolution TEM (HRTEM) images and SAED patterns are also presented. Regardless of deposition conditions, a crystalline and smooth film uniformly deposited on thermally oxidized silicon substrate is obtained even under such a short deposition time of only 45 s; additionally, their morphologies also reveal a very dense almost glassy structure. Apparently, these features are attributed to the advantages of high density plasma and high ionization rate for HIPIMS plasma discharge, resulting in intensified ion bombardment during film growth^16–18^. The thickness of the TiO_x_ films prepared at the oxygen flow rate of 15, 17.5 and 20 sccm are estimated to be approximately 16.2, 13.5, and 12.3 nm, respectively, i.e., the growth rate slightly decreased with increase in oxygen flow rate. Further, the TEM-SAED technique was used to characterize the crystal structure of the HIPIMS-TiO_x_ films, as shown at the right side of each BF images in Figs 1–3. All SAED patterns consist of distinct spots, indicating a well-defined polycrystalline structure. Upon close inspection of SAED patterns, we can find that both metallic Ti and γ-TiO phases are co-existing in the TiO_x_ film obtained under the oxygen flow rate of 15 sccm as evidenced from the lattice spacing (Fig. 1), whereas diffraction pattern from pure γ-TiO phase were detected at the oxygen flow rate of 17.5 sccm (Fig. 2). Furthermore, the mixed-phase of γ-TiO and rutile (R-TiO_2_) for the TiO_x_ film is found at relative high oxygen flow rate of 20 sccm (Fig. 3). These results demonstrated that in preparing the HIPIMS-TiO_x_ coatings, adjusting the oxygen flow rate during deposition enabled the control of the phase structure to attain γ-TiO and R-TiO_2_ phase successfully.

**Figure 4:**
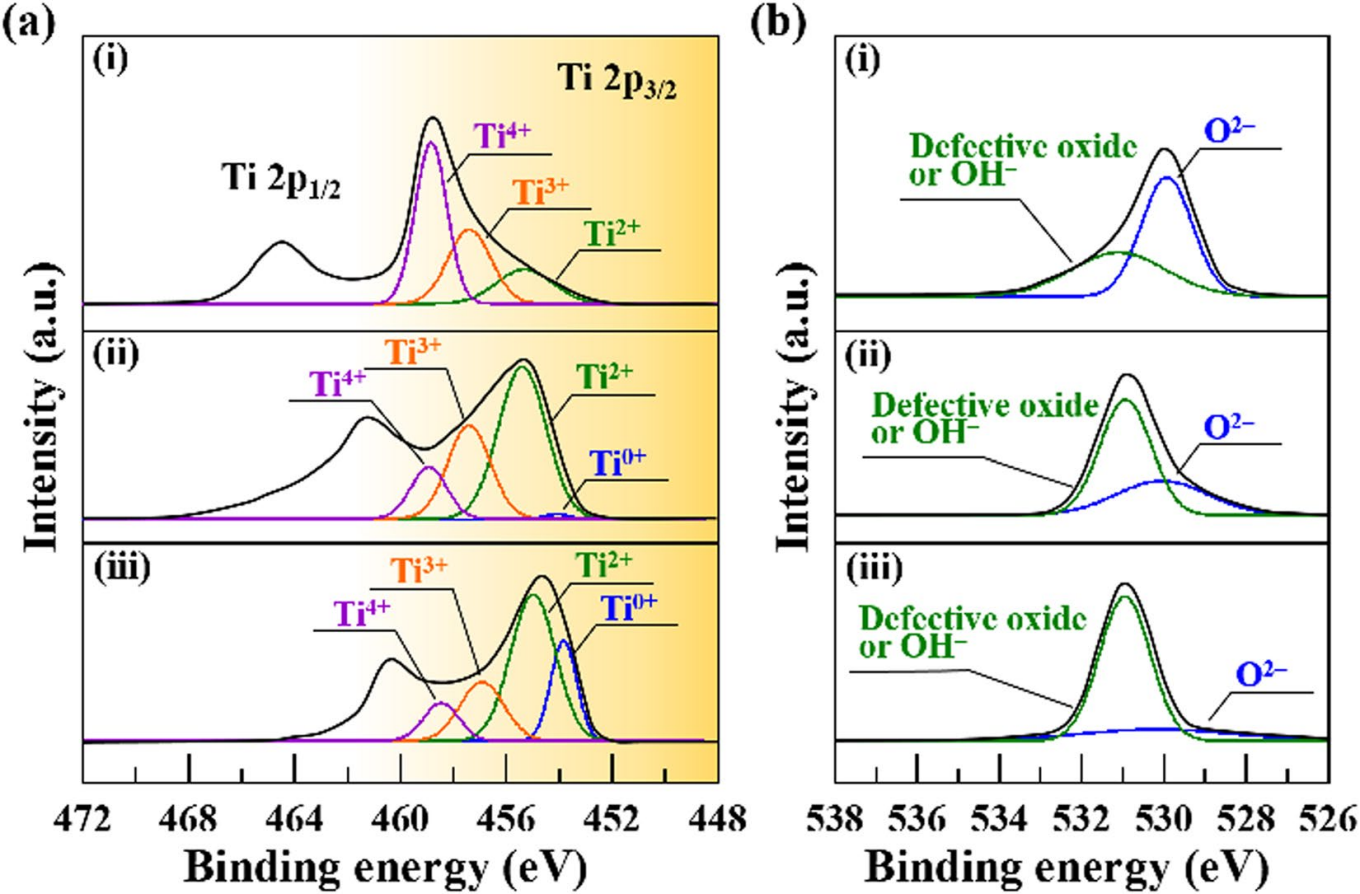
Detailed (**a**) Ti 2p and (**b**) O 1 s XPS spectra of the TiO_x_ films deposited by HIPIMS under different oxygen flow rates, (i) 20 sccm, (ii) 17.5 sccm, and (iii) 15 sccm.

**Figure 5:**
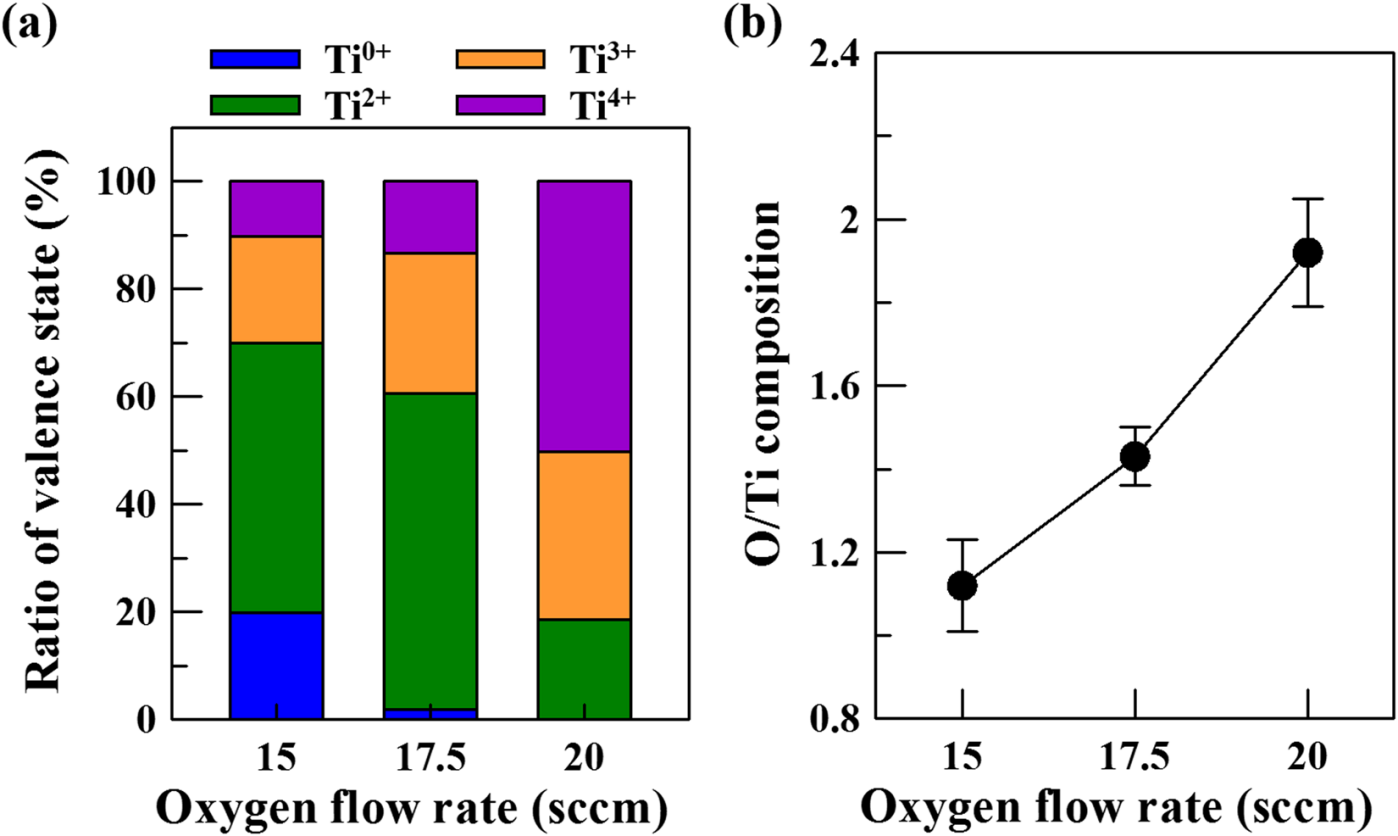
(**a**) Ratio of valence state and (**b**) O/Ti composition of the TiO_x_ films deposited by HIPIMS under different oxygen flow rates.

Detailed Ti 2p and O 1 s XPS spectra of the TiO_x_ films deposited by HIPIMS at different oxygen flow rates are revealed in Fig. 4. To avoid the influence of surface oxide impurities on the XPS result, the XPS measurement was performed at 6 nm depth from sample surface. The Ti 2p_3/2_ spectra can be deconvoluted by assuming contribution from the Ti°, Ti^2+^, Ti^3+^ and Ti^4+^ as shown in Fig. 4a, indicating the features associated with several valence states of Ti that exist in the films^19–21^. The signal of Ti° at around 453~454 eV is attributed to the metallic state of Ti; Ti^2+^ (~455.1 eV) and Ti^3+^ (~457.8 eV) are ascribed to the TiO and titanium suboxide, respectively; the Ti^4+^ near 458.7 eV is designated as TiO_2_^19–21^. By examining the deconvoluted spectra (colored curves) of the TiO_x_ films obtained at different oxygen flow rates, the intensity of the peaks that attribute to higher valence is apparently increased with increasing oxygen flow rate *i.e*., the bonding energy of the Ti was increased. The ratio of valence state for the obtained TiO_x_ films were further quantified according to their integrated intensity, as shown in Fig. 5a. The results show that the TiO_x_ film obtained under the oxygen flow rate of 15 sccm is mainly composed of Ti^0^ and Ti^2+^, namely metallic Ti and bi-valence state of Ti of γ-TiO. At the oxygen flow rate of 17.5 sccm, the dominant bonding structure for the obtained TiO_x_ film is Ti^2+^. When the oxygen flow rate was further increased to 20 sccm, a predominant valence state of Ti^4+^ (viz. TiO_2_ phase) exists in the obtained TiO_x_ film. Consequently, the results of XPS analysis in the obtained TiO_x_ film agree well with the findings in TEM observation as shown in Figs 1–3. Overall to say, across the range of substrate bias voltage investigated in this study, multiple-valence titanium oxides are found in the obtained films. On the other hand, as can be seen, the O 1 s XPS spectra shown in Fig. 4b present that the trend in bi-valence oxygen and defective oxides or absorptive OH^–^ follow the valence states of Ti^19,21^.

Linking the results of TEM observation and XPS analysis, a short summary of microstructure for the deposited TiO_x_ film can be drawn clearly. For the film prepared at the oxygen flow rate of 15 sccm, the quite a number of metallic titanium state exists in γ-TiO structure, indicating the mixed-phase of Ti and γ-TiO in the film. When the oxygen flow rate increased to 17.5 sccm, the intensity of Ti^2+^ augmented significantly, showing the pure γ-TiO phase in the film. Over an oxygen flow rate of 20 sccm, the R-TiO_2_ is the main crystal structure with minor γ-TiO phase in the obtained TiO_x_ film.

Figure 5b shows the O/Ti composition of the TiO_x_ films deposited by HIPIMS under different oxygen flow rates. The experimental error ranges (error bars) of O/Ti ratios for these three TiO_x_ films are added in Fig. 5b. Based on the TEM and XPS results, the film obtained at the oxygen flow rate of 15 sccm is identified to be mixed-phase of Ti and γ-TiO, which have a stoichiometric ratio of O/Ti of 1.12. For the TiO_x_ film deposited at oxygen flow rate of 17.5 sccm, the O/Ti ratio increase to 1.43. Interestingly, this film still have a stable γ-TiO structure, even with such an over-saturated O/Ti ratio. The mechanism behind the formation of oxygen supersaturated γ-TiO by using HIPIMS discharge is reported in our previous study^16^. According to the literature^15^, γ-TiO is with structural vacancies that occur due to the substitution of atoms by structural vacancies in one of the sublattices. Moreover, γ-TiO exhibits a high amount of structural vacancies up to 15 at.% on both Ti and O sublattices. Since the oxygen supersaturated γ-TiO film obtained at an oxygen flow rate of 17.5 sccm in our case, structural vacancies shall occur to the Ti sublattices. When the oxygen flow rate is further increased to 20 sccm, the O/Ti ratio in the obtained TiO_x_ film is 1.92, which presents an O-inadequate R-TiO_2_ crystal structure.

**Figure 6:**
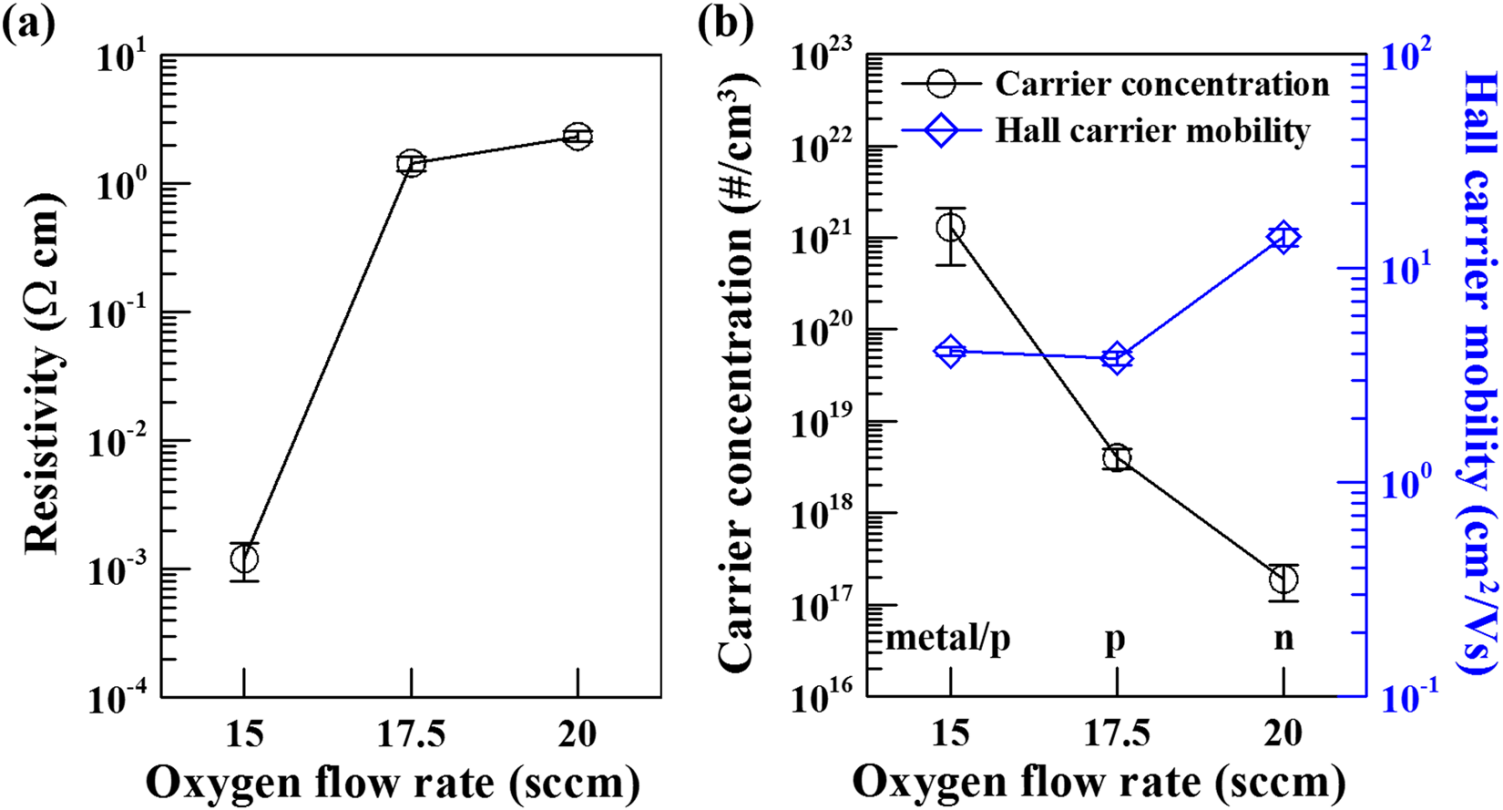
Electrical properties in terms of (**a**) resistivity, and (**b**) carrier concentration and carrier mobility of the TiO_x_ films deposited by HIPIMS under different oxygen flow rates.

Figure 6 shows the electrical properties of the TiO_x_ films deposited by HIPIMS under different oxygen flow rates. Electrical properties of TiO_x_ films are average values via three-point calculation, and the standard deviations (error bars) are also shown in Fig. 6. Clearly, the variation of electrical properties (in term of resistivity, hall carrier concentration and carrier mobility) for the obtained TiO_x_ films depend strongly on the transition between various phases. Films deposited at low oxygen flow rate (i.e. 15 sccm) has lots of metallic Ti content in γ-TiO structure, which results low resistivity (1.2 × 10^−3^ Ω∙cm) and high carrier concentration (1.3 × 10^21^ #/cm^3^) in the obtained TiO_x_ film. In addition, this film also showed the fluctuations in the sign and magnitude of hall coefficient during Hall effect measurement. Therefore, at oxygen flow rate of 15 sccm, the obtained TiO_x_ film exhibits metallic and weak p-type characteristics. At sufficient oxygen flow rate condition, titanium gets oxidization initially to γ-TiO phase and then to TiO_2_ phase with further increase in oxygen flow, resulting in the formation of semiconducting materials and increase in resistivity. Under the preparation condition of oxygen flow rate of 17.5 sccm, the carrier concentration of the obtained TiO_x_ film with pure γ-TiO phase is still high, 4.0 × 10^18^ #/cm^3^, because of the large amount of vacancies in the Ti sublattices. As a result, this TiO_x_ film possesses p-type characteristics. Conversely, for the film obtained at oxygen flow rate of 20 sccm which has a prominent O-inadequate R-TiO_2_ structure, the charge carrier can be considered originating from oxygen vacancies, subsequently forming n-type semiconductor. It is also found that the carrier concentration was decreased for the TiO_x_ film obtained at oxygen flow rate of 20 sccm. This is due to the transition zone from γ-TiO to R-TiO_2_. On the other hand, the obtained TiO_x_ films exhibited the hall carrier mobility of 3.8 cm^2^/Vs and 14.0 cm^2^/Vs for p-type γ-TiO phase and n-type TiO_2_ phase, respectively in this study. When the Ti ion has a high valence (i.e., Ti^4+^), it will produce the n-type TiO_2_ film. However, as the Ti ion valence of the TiO_x_ is mainly divalent, it will become the p-type film with the γ-TiO structure. It is well-known that the carrier mobility exhibits a strong relationship with crystallinity and carrier concentration of material. In addition, the valence state is also a probable factor to change the electronic band structure^22^, resulting in the change on the mobility. In this study, the HIPIMS technique was used to prepare TiO_x_ thin films. Because the HIPIMS technique has both the high-density plasma and the high ion bombardment energy, it can grow high-quality thin films at low temperatures. Even if the deposition time is only 45 s, crystalline γ-TiO and TiO_2_ films both can be prepared, which will enable the TiO_x_ film to have a higher carrier mobility.

After realizing the conditions to get p-type and n-type characteristics in the obtained TiO_x_ films, TiO_x_-based TFTs with staggered bottom gate configuration were fabricated. Based on the abovementioned results, deposition of TiO_x_ films as channel layers in the TFT devices were carried out at the oxygen flow rate of 17.5 sccm for γ-TiO and 20 sccm for TiO_2_. Additionally, the film thickness of channel layers for γ-TiO and TiO_2_ were 13.5 nm and 12.3, respectively.

**Figure 7:**
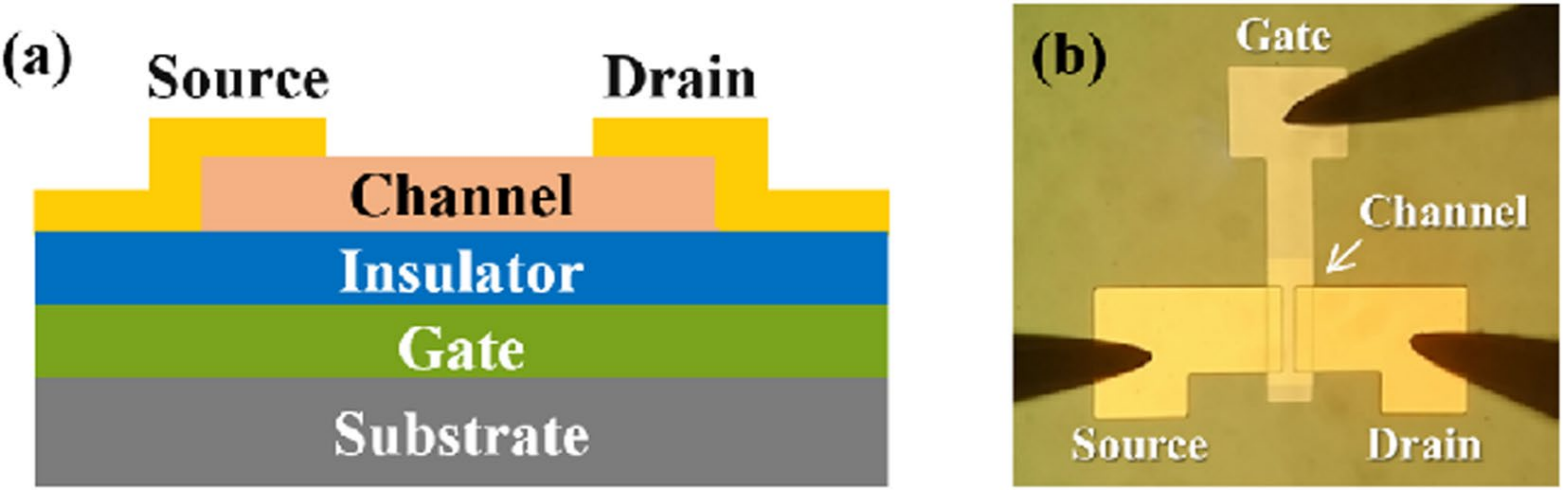
(**a**) Schematic cross-sectional image and (**b**) optical top-view micrograph of the TiO_x_-based TFT with staggered bottom gate configuration.

**Figure 8:**
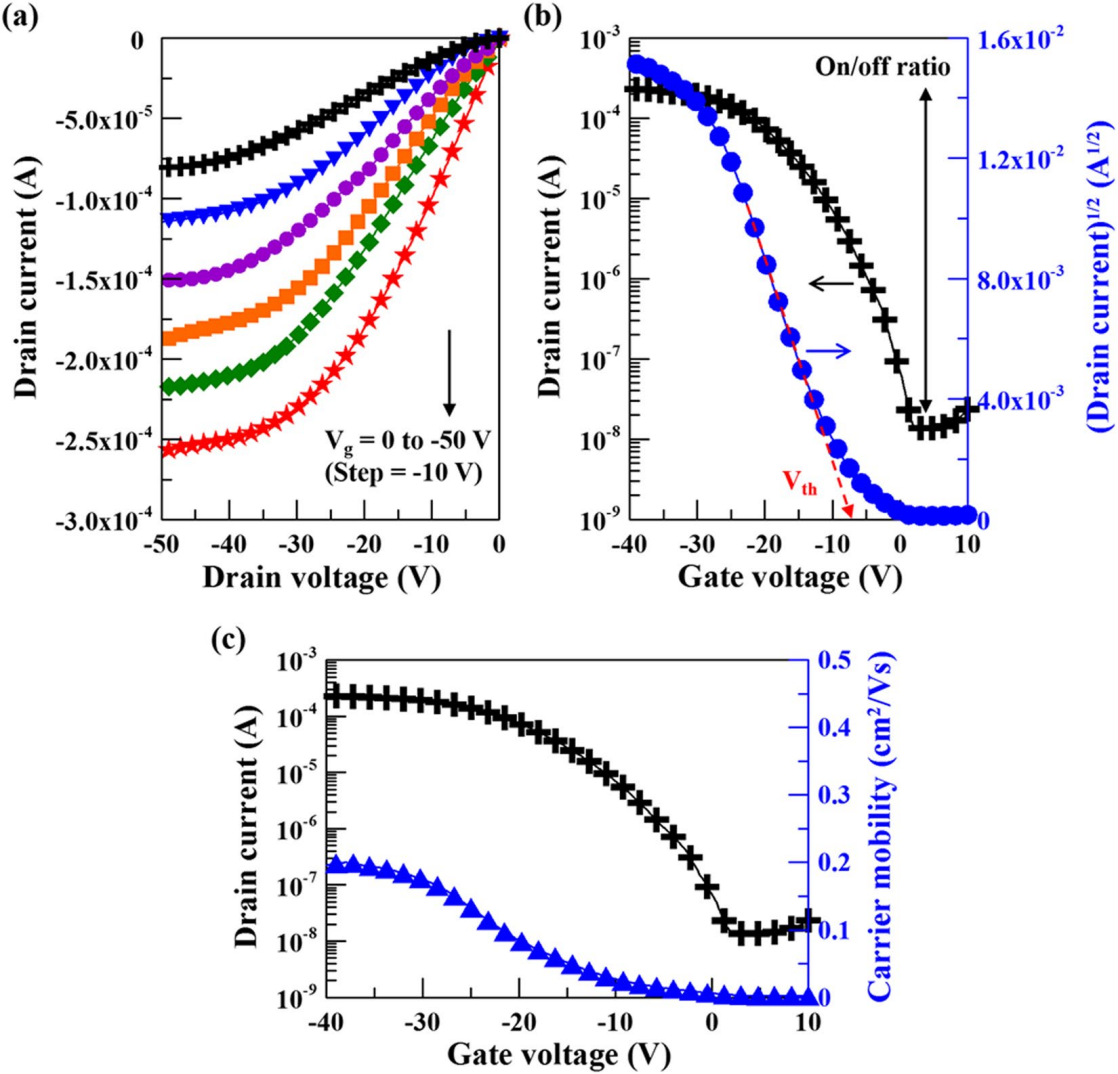
(**a**) Output characteristics, (**b**) transfer characteristics and (**c**) corresponding carrier mobility curves of the p-type γ-TiO TFTs. Estimation of on/off ratio and threshold voltage are also shown on corresponding transfer curves.

**Figure 9:**
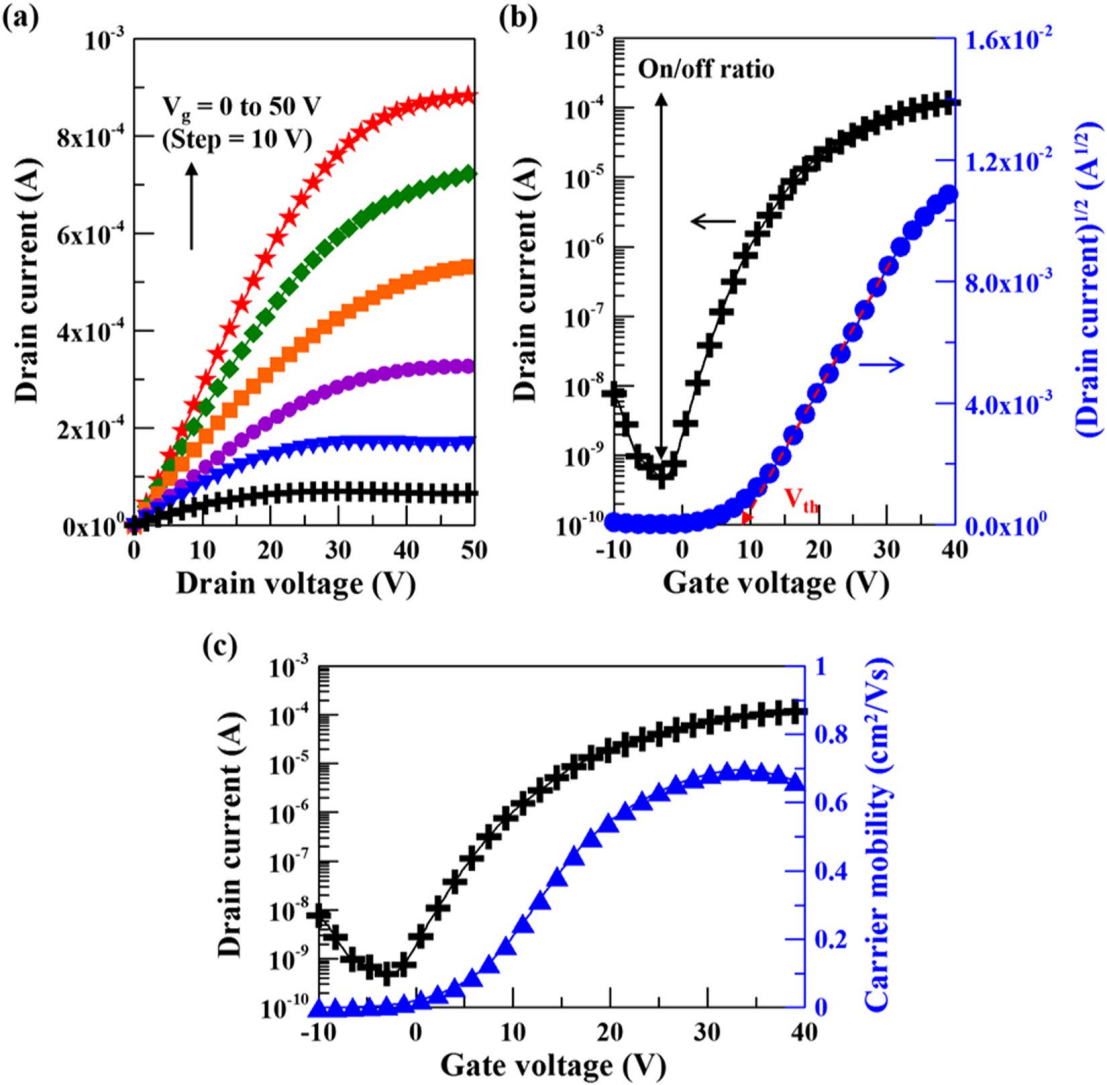
(**a**) Output characteristics, (**b**) transfer characteristics and (**c**) corresponding carrier mobility curves of the n-type TiO_2_ TFTs. Estimation of on/off ratio and threshold voltage are also shown on corresponding transfer curves.

The optical top-view micrograph and the schematic cross-sectional image of the TiO_x_-based TFT with staggered bottom gate configuration are shown in Fig. 7 (in the Methods part). Figures 8 and 9 show the electrical properties in terms of output characteristics, transfer characteristics and corresponding carrier mobilities of γ-TiO and TiO_2_ channel TFTs, respectively. Both output characteristic curves showing linear relationship at low source–drain voltages (V_d_), as depicted in Figs 8a and 9a, illustrate the effect of contact resistance is not detected. This suggests high interface integrity in γ-TiO and TiO_2_ TFTs. γ-TiO channel TFT shows typical p-type field effect transistor behavior and TiO_2_ channel TFT shows typical n-type field effect transistor behavior. Moreover, both types of TiO_x_-based TFTs operated in enhancement mode. The transfer characteristics of the γ-TiO channel p-type TFT at a constant V_d_ = −30 V, as revealed in Fig. 8b, indicates an off-current (I_off_) of 1.5 × 10^−8^ A, an on-current (I_on_) of 2.5 × 10^−4^ A, corresponding an on/off current ratio, I_on_/I_off_, of 1.7 × 10^4^. For the TiO_2_ channel n-type TFT, the performance extracted from the transfer characteristics at a constant V_d_ = 30 V (Fig. 9b) shows an I_off_ of 4.8 × 10^−10^ A and an on-current (I_on_) of 1.2 × 10^−4^ A, as well as an I_on_/I_off_ of 2.5 × 10^5^. Furthermore, by deducing from the transfer characteristics, the field-effect carrier mobility (μ_FE_) and threshold voltage (V_th_) were, respectively, 0.2 cm^2^/Vs (Fig. 8c) and −7.1 V for p-type γ-TiO TFT, and 0.7 cm^2^/Vs (Fig. 9c) and 8.8 V for n-type TiO_2_ TFT. As mentioned earlier, the n-type oxide semiconductor exhibits the highly spherical symmetric metal *ns* orbital, and results in the less carrier effective mass^6,7^. So, p-type γ-TiO TFT has low carrier mobility compared to their n-type counterpart TiO_2_.

In brief, the feasibility of synthesize TiO_x_ films with both types of conduction characteristics by varying the oxygen flow rate during the HIPIMS deposition were achieved. Furthermore, based on our best knowledge, this is the first article reporting use of γ-TiO film as channel layer for p-type TFT device. μ_FE_ accompanies I_on_/I_off_ of 0.2 cm^2^/Vs and 1.7 × 10^4^, respectively, were obtained for p-type γ-TiO TFT; in addition, n-type TiO_2_ TFT exhibits the μ_FE_ of 0.7 cm^2^/Vs and I_on_/I_off_ of 2.5 × 10^5^. Table 1 shows a summary of the TiO_2_ or TiO_x_-based TFTs prepared by various techniques and their related characteristics. These TiO_2_ or TiO_x_ films were prepared by metalorganic chemical vapor deposition (MOCVD), ALD, sputtering (DC or RF), and solution methods. The conductive types and mobilities of these films are compared. Additionally, the on/off current ratios of TFT devices fabricated with these films are also summarized. As mentioned above, these TiO_2_ or TiO_x_-based TFTs are all n-type devices. Obviously, the device performances of our TFTs possess the similar level in comparison to those of other researches. Furthermore, our results confirm that the practically semiconductor and optoelectronic applications can be expanded significantly through the TiO_2_/TiO growth by HIPIMS. This is attributed to its tunable conductive type via the adjustment of oxygen flow rate. Why the highly stable p-type γ-TiO TFT with good quality can be fabricated by HIPIMS? Actually, during the sputtering and CVD processes, the oxygen flow rate can also be adjusted for the TiO_2_/TiO growth. However, the stable p-type TiO_x_ films have never been prepared via these methods. When the TiO_2_/TiO is deposited by sputtering or CVD, the high-temperature growth condition is required to obtain the high-quality film. Besides, the high-temperature process is helpful to growing the TiO_2_ or Ti_2_O_3_ film through the bonding between Ti and O atoms. In other words, the TiO_2_ or Ti_2_O_3_ with n-type conductivity is the more stable crystal phase under high-temperature growth condition. Even under the oxygen-poor condition, the sputtered or CVD-grown film possibly belongs to TiO_2_(Ti_2_O_3_)-TiO mixed oxides, and its p-type conductivity cannot appear. It is worth mentioning the stable γ-TiO films are more easily formed under low-temperature and oxygen-poor conditions. Therefore, the HIPIMS technique is indeed suitable for the growth of p-type TiO_x_ films. Most importantly, the high power generated by HIPIMS can provide enough energy for Ti and O atoms to enhance the quality of γ-TiO film (not similar to the thermal energy generated by high-temperature process), improving the mobility of hole carrier. This is the reason why the highly stable p-type γ-TiO TFT can be achieved by HIPIMS.

**Table 1:** Summary of the TiO_2_ or TiO_x_-based TFTs prepared by various techniques and their related characteristics.

Growth method	Conductive type	Mobility (cm^2^/Vs)	On/off current ratio	Ref.
RF sputtering	n-type	10.7	10^4^	12
RF sputtering	n-type	0.03	1.45 × 10^2^	23
RF sputtering	n-type	0.01	10^4^	24
RF sputtering	n-type	0.11	3.4 × 10^5^	25
DC sputtering	n-type	0.002	10^4^	26
MOCVD	n-type	0.063	2.7 × 10^5^	27
ALD	n-type	0.014	4.3 × 10^5^	28
ALD	n-type	0.47	10^5^	29
Solution method	n-type	0.239	3.85 × 10^5^	30
HIPIMS	n-type	0.7	2.5 × 10^5^	This study
	p-type	0.2	1.7 × 10^4^	

## Conclusion

Both p-type γ-TiO and n-type TiO_2_ films were prepared by using HIPIMS deposition with carful control of oxygen flow rate, and their applications on TFTs were also developed. For common deposition methods such as sputtering or CVD, the high-temperature growth process or additional post-thermal treatment is required to achieve device-quality TiO_x_ films. However, the high-temperature condition is indeed an obstacle for obtaining the stable p-type γ-TiO film. This is attributed that the TiO_2_ or Ti_2_O_3_ with n-type conductivity is the more stable crystal phase under high-temperature growth condition. Fortunately, the HIPIMS technique with both very high ionization level and power energy can produce the high-quality TiO_x_ films under low-temperature growth condition, which makes it possible to prepare the highly stable p-type γ-TiO film via the suitable adjustment of oxygen flow rate. In this study, the γ-TiO and TiO_2_ films can be deposited at oxygen flow rates of 17.5 and 20 sccm, respectively. For the γ-TiO film, the p-type conductivity arises from the structural vacancies in the Ti sublattices; on the other hand, the n-type conductivity in the TiO_2_ phase is due to the formation of O-inadequate R-TiO_2_ in the obtained film. Furthermore, the μ_FE_ of TFTs fabricated with p-type γ-TiO and n-type TiO_2_ channels are 0.2 and 0.7 cm^2^/Vs, respectively. Meanwhile, the I_on_/I_off_ ratios of these two devices are 1.7 × 10^4^ and 2.5 × 10^5^, respectively. Consequently, two major breakthroughs could be further achieved based on the first proposed p-type γ-TiO device. First, the p-n combinational devices (such as CMOSs and p-n junction optoelectronic devices) will be carried out more efficiently. Secondly, by properly tuning the oxygen flow rate during HIPIMS growth, various oxide materials with both p- and n-type conductivities also can be prepared.

## Methods

### Preparation and Characteristics of titanium oxide films

Thermally oxidized silicon wafer used as substrates, were ultrasonically cleaned prior to the deposition work. The HIPIMS apparatus is described in detail elsewhere^16^, while titanium metal served as cathode, and argon/oxygen served as sputtering/reactive gases. After the coating chamber was evacuated to a base pressure of 6 × 10^−3^ Pa, the argon plasma pre-treatment was applied for removing surface contamination. During HIPIMS deposition, the varied oxygen flow rates were adjusted to control the crystal structure of TiO_x_ film under a fixed pressure of 0.133 Pa (1 mTorr), argon flow rate of 100 sccm, and pulse-DC substrate bias voltage of −50 V for 45 s. The detailed HIPIMS output power parameters and deposition conditions used in this study are shown in Table 2.

**Table 2:** Deposition conditions for preparing TiO_x_ films.

Deposition Parameters	Process Data		
Working pressure (Pa)	0.133		
Argon flow rate (sccm)	100		
Discharge frequency (Hz)	800		
Duty cycle, T_on_/T_off_ (μs)	150/1250		
Discharge voltage (V)	800		
Deposition time (s)	45		
Substrate bias voltage (−V)	50		
Argon flow rate (sccm)	100		
Oxygen flow rate (sccm)	15	17.5	20
Peak discharge current (A)	~120	~130	~140
Peak discharge power (kW)	~4600	~4700	~4900

The crystallographic structure of the deposited TiO_x_ films was identified using transmission electron microscopy (TEM, JEOL JEM-2010) to obtain the cross-sectional bright field (BF) images of the whole film and the selected area electron diffraction (SAED) patterns at 200 kV. An ULVAC-PHI 5000 VersaProbe scanning XPS microprobe instrument was used to obtain X-ray photoelectron spectra (XPS), where the monochromatic Al Kα (1486.71 eV) was employed to characterize bonding energy of the Ti 2p and O 1 s core electron for identifying the respective Ti and O chemical state in the TiO_x_ films. Elemental composition of the obtained TiO_x_ films was also quantified. All XPS spectra of the samples were fitted well with Gaussian functions. The transmission function, effective cross-section, escape depth, and inelastic mean free path for photoelectrons were used for quantification. The electrical properties in terms of resistivity, hall carrier mobility (μ_hall_) and carrier concentration (n_e_) of the obtained TiO_x_ films was carried out by using a Hall effect measurement system (Ecopia HMS-3000) with van der Pauw method.

### Preparation and characteristics of titanium oxide-based thin film transistor**s**

In this study, the staggered bottom gate configuration was adopted to fabricate TiO_x_-based TFT devices, owing to its advantages of easier processing and enhanced electrical properties^1^. After the thermally oxidized silicon wafer was ultrasonically cleaned in a series of acetone, isopropanol, and deionized water baths, a thermal-evaporated Ti (10 nm)/Al (50 nm)/Ti (10 nm)/Au (60 nm) film was deposited followed by the lift-off process to make the patterned gate electrode. Atomic layer deposition (ALD) was sequentially used to deposit a 100 nm-aluminum oxide film for gate dielectric layer using trimethylaluminium (TMA) and water as precursors. Then a HIPIMS-TiO_x_ film (viz. γ-TiO or TiO_2_ film) was grown according to the aforementioned conditions as the channel material; afterward the patterned channel layer was realized by photolithography, and inductively coupled plasma-reactive ion etching (ICP-RIE) processes. Finally, the source and drain electrodes were prepared by using thermal-evaporated Ti (10 nm)/Al (50 nm)/Ti (10 nm)/Au (60 nm) film, and then defined by lift-off process. The working pressure and growth temperature of Ti/Al/Ti/Au were fixed at 6 × 10^−7^ Torr and 40 °C, respectively. After evaporating the Ti/Al/Ti/Au film, the ohmic contact was formed (without the post-annealing process). In the present work, the channel length and width were set to be 20 μm and 100 μm, respectively. The schematic cross-sectional image and the optical top-view micrograph of the TiO_x_-based TFT with staggered bottom gate configuration are shown in Fig. 7. Electrical properties in terms of output characteristics and transfer characteristics of the TiO_x_-based TFTs were measured using a probe station with a semiconductor parameter analyzer (Keithley 4200-SCS). The field-effect carrier mobility (μ_FE_), on/off current ratio (I_on_/I_off_), and threshold voltage (V_th_) were then estimated.
